# Outcomes of first line chemotherapy in patients with chronic lymphocytic leukemia

**DOI:** 10.12669/pjms.325.10480

**Published:** 2016

**Authors:** Adil Nazir, Fawad, Sheeraz Ali, Farhana Badar, Neelam Siddique, Abdul Hameed

**Affiliations:** 1 Dr. Adil Nazir, MBBS, FCPS Fellow Medical Oncology, Department of Medical Oncology, Shaukat Khanum Memorial Cancer Hospital & Research Centre, Lahore - Pakistan; 2 Dr. Fawad MBBS, FCPS Fellow Medical Oncology, Department of Medical Oncology, Shaukat Khanum Memorial Cancer Hospital & Research Centre, Lahore - Pakistan; 3 Dr. Sheeraz Ali, MBBS, FCPS Fellow Medical Oncology, Department of Medical Oncology, Shaukat Khanum Memorial Cancer Hospital & Research Centre, Lahore - Pakistan; 4 Dr. Farhana Badar, Sr. Biostatistician & Cancer Epidemiologist Cancer Registry & Clinical Data Management, Department of Cancer Registry and Clinical Data Management; 5 Dr. Neelam Siddiqui, MBBS, FRCP (Glasgow), CCST (Medical Oncology) UK, Consultant medical oncologist, Department of Medical Oncology, Shaukat Khanum Memorial Cancer Hospital & Research Centre, Lahore - Pakistan; 6 Dr. Abdul Hameed MBBS, MD, FRCP (Edin), Consultant Hematologist, Department of Medical Oncology, Shaukat Khanum Memorial Cancer Hospital & Research Centre, Lahore - Pakistan

**Keywords:** Chronic lymphocytic leukemia (CLL), Chemotherapy

## Abstract

**Objective:**

Chronic lymphocytic leukemia (CLL) is a heterogeneous disease in terms of survival with and without treatment. Many chemo and immunotherapeutic agents are available to treat this indolent disease. Aim of this study was to determine the outcomes of patients with chronic lymphocytic leukemia treated with different available chemotherapeutic regimens.

**Methods:**

All patients with diagnosis of CLL from 2008 to 2013 were included. Data were collected from hospital information system. Objective response rate (ORR) in terms of complete or partial response (CR, PR), stable or progressive disease (SD, PD), overall survival (OS), and progression free survival (PFS) were calculated.

**Results:**

Fifty seven patients were included; 42 (74%) male and 15 (26%) were female. Patients with Binet stage A 10 (18%); B 20 (35%) and C were 27(47%). Median age was 50.9 years. Forty six (80%) were treated and 11(20%) remained on watch and wait. Treatment indications were B symptoms 14 (30%), symptomatic nodal disease 18(39%), thrombocytopenia 4(9%), anemia 7(15%) and doubling of lymphocyte count 3 (7%). Chemotherapy regimens used were FC in 38 (83%), FCR 5(11%), chlorambucil 2(4%) and CVP in 1(2%) patient. Twenty two (56%) patients had CR, 13(33%) PR, 3(7.6 %) SD, and 1(2.5%) had PD. ORR was 89%. Median PFS was 23.1 months and median 3 years OS was 55%.

**Conclusion:**

Majority of patients was in a relatively younger age group and presented with advanced stage disease requiring treatment. Small number of patients received rituximab due to cost. PFS and OS are comparable with published literature.

## INTRODUCTION

CLL is a disease of elderly, with male predominance and a median age of more than 70 years. Median age is 58 years in familial cases.^1^ CLL is an incurable disease with a remitting and relapsing course and life-long observation and follow-up is recommended. There are two staging systems, Binet and Rai, separating patients with different prognoses.^2^,^3^ Overall survival (OS) of patients with advanced disease has improved with the new treatment options.^4^ Previous studies have shown that treatment with chemotherapeutic agents does not translate into a survival advantage in patients without symptoms and an early-stage disease.^5^ The standard treatment of asymptomatic is a watch-and-wait strategy. Treatment should only be initiated in patients with active disease that includes B symptoms, cytopaenias, symptoms or complications from lymphadenopathy, splenomegaly or hepatomegaly, lymphocyte doubling time of <6 months, autoimmune anaemia and/or thrombocytopenia non responsive to steroids.^6^

In physically fit patients FCR (Fludarabine, Cyclophosphamide, Rituximab) is the standard first-line therapy.^7^ Combinations based on other purine analogues such as cladribine^8^ or pentostatin^9^ have shown similar activity, but it is uncertain whether they can replace fludarabine in FCR regimen. In elderly, FCR is associated with a higher rate of severe infections than bendamustine plus rituximab (BR). However, BR produces fewer complete remissions than FCR.^10^ In patients with comorbidities and older age, combination of chlorambucil plus an anti-CD20 antibody (rituximab, ofatumumab or obinutuzumab) is considered as standard approach.^11^,^12^

In this study, we analyzed objective response rate, progression free survival and overall survival in patients with chronic lymphocytic leukemia treated with different available chemotherapeutic regimens.

## METHODS

This is a retrospective study. All patients of CLL from October 2008 to September 2013 were studied. Diagnosis was made according to standard guidelines.^13^ Patient characteristics such as age, gender, hemoglobin (Hb), lymphocyte count, white blood cells, platelet count, bone marrow biopsy and CT scan results (before and after treatment) were analyzed. Binet system was used for staging. Indications for treatment^6^ and type of chemotherapy regimens were noted.

Complete and partial response (CR, PR), stable disease (SD), progressive disease (PD) and relapse were defined according to National Cancer Institute–Working Group 1996 guidelines.^14^ PFS defined as time from start of the treatment to disease progression or death. OS defined as time from enrollment of patient in hospital to death from any cause or last follow up.

### Statistical analysis

Distributions were determined as frequencies and percentages for categorical variables. For continuous variables mean, median, standard deviation and range were computed. Progression free interval survival, interval between dates of treatment ended and relapse in months, was conducted using the Kaplan *Meier* method and the end point of interest was relapse. The log rank test was applied and considered to be significant at an alpha level of 0.05. The analysis was conducted using SPSS, version 19.

## RESULTS

**Fig.1:**
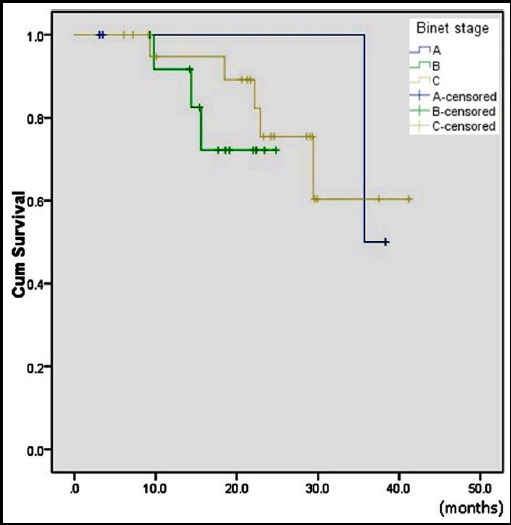
Kaplan-Meier survival curve for progression free survival according to stage.

**Fig.2:**
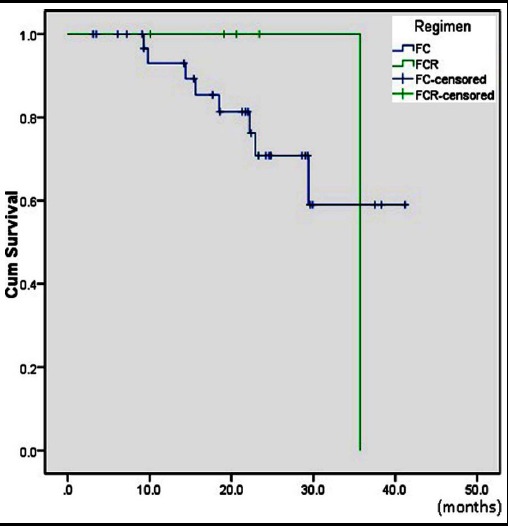
Kaplan-Meier survival curve for progression free survival according to regimens.

Fifty seven patients were included. Forty two (74%) patients were male and 15(26%) were female. Median age was 50.9+8.2 years (range 25-72). Patients of Binet stage A, B and C; were 10(18%), 20 (35%) and 27(47%), respectively. Forty six (80%) patients were treated and 11(20%) remained on watch and wait policy. Treatment indications were B symptoms in 14(30%), bulky disease 18(39%), thrombocytopenia 4(9%), anemia 7(15%) and doubling of lymphocyte count in 3 (7%) patients. Chemotherapy regimens used were FC in 38 (83%), FCR 5(11%), CVP 1(2%) and chlorambucil in 2(4%) patients (Table-I). Two patients developed acute kidney injury (AKI), one patient died before any intervention and one patient lost to follow up. These four patients were excluded. Three patients treated with chlorambucil (2) or CVP (1) were also excluded. Twenty two patients (56%) achieved CR, 13(33%) PR, 3(7.6 %) SD, and 1(2.5%) had PD (Table-II). Overall response rate (ORR) was 89%. There were 9 (23.9 %) patients who had disease relapse. More relapses were seen in FC group 8(23.5%) compared to FCR 1 (20%). Median PFS for whole group was 23.1 months and median three years OS was 55%. However, PFS was significantly higher for stage A (35.7 months) compared to B (14.4 months) and C (22.2 months), p=0.05 (Table-III, Fig.1). In addition, PFS was better in FCR group than FC, p=0.04 (Table-III, Fig.2). Due to small number of patients, both regimens were not compared for survival analysis.

**Table-I:** Characteristics, stage and treatment.

*Age (years)*	*Median 50.9±8.2 (range 25-72)*
*Gender*	*n=*

Males	42(74%)
Females	15(26%)

*Binet’s stage*	*n=*

A	10(18%)
B	20 (35%)
C	27(47%)

*Lymphocyte count (103/microlitre)*	*Mean 107.56 (range 4-466)*

*Presentation*	*n=*

B symptoms	14(30%)
Bulky disease	18(39%)
Thrombocytopenia	4(9%)
Anemia	7(15%)
Doubling lymphocyte count	3 (7%)

*Management*	*n=*

Treated	46(80%)
Watch and wait	11(20%)

*Chemotherapy*	*n=*

FC	38 (83%)
FCR	5(11%)
Chlorumbucil	2(4%)
CVP	1(2%)

**Table-II:** Responses according to stage and regimen.

*Response*	*Regimen*	*Binet’s Stage*			*Total treated*

		*A*	*B*	*C*	*Patients n=39*
CR	FC	1(2.5%)	6(15.3%)	12(30.7%)	19(48.7%)
	FCR	-	-	3(7.6%)	3(7.6%)
	Total	1(2.5%)	6(15.3%)	15(38.4%)	22 (56.4%)
PR	FC	3(7.6%)	5(12.8%)	3(7.6%)	11(2.8%)
	FCR	-	-	2(5.1%)	2(5.1%)
	Total	3(7.6%)	5(12.8%)	5(12.8%)	13 (33.3%)
SD	FC	-	1(2.5%)	2(5.1%)	3(7.6%)
	FCR	-	-	-	
	Total	-	1(2.5%)	2(5.1%)	3 (7.6%)
PD	FC	-	-	1(2.5%)	1(2.5%)
	FCR	-	-	-	-
	Total	-	-	1(2.5%)	1 (2.5%)

**Table-III:** Follow up of CLL patients according to stage and chemotherapy regimen.

*Relapse*	*Binet stage*	*N*	*Median (months)*	*p value*
Yes	A	1	35.7	0.05
	B	3	14.4	
	C	5	22.2	
	Total	9	18.2	
No (On follow up)	A	3	3.5	0.31
	B	10	18.8	
	C	17	24.2	
	Total	30	21.8	

*Relapse*	*Regimens*	*N*	*Median (months)*	*P value*

Yes	FC	8	17.0	0.04
	FCR	1	35.7	
	Total	9	18.5	
No (On follow up)	FC	26	22.2	0.59
	FCR	4	198	
	Total	30	21.8	

## DISCUSSION

Outcomes of CLL are variable, some patients have very indolent course while other may have aggressive disease behaviour from the start.^15^ A significant number of patients may not need treatment at the time of presentation and they could be put on observation.^16^ Treatment is started when patients develops symptoms or cytopenias, which are disease related. The treatment paradigm for CLL has changed significantly over the last few years.^17^

There is a strong evidence that FCR is better than FC, however, former is associated with more side effects.^7^,^18^ A systematic review with meta-analysis of clinical trials between 2000 and 2012 compared FC and FCR in patients with CLL. PFS and OS showed significant difference between two regimens, with CR more frequent with FCR but significantly higher neutropenia and serious adverse reactions.^19^

Our results showed that the onset of CLL is in a relatively younger age group compared to western data.^1^ One explanation may be due to shorter life span in our region. CLL was common in males as expected. The vast majority of patients needed treatment at the time of diagnosis and this is consistent with published literature that younger patients have aggressive disease behaviour and shorter time to first treatment.^20^ The majority of patients presented with advanced stage disease (Binet stage B&C). That may be another reason to start early treatment. One reason for this could be delay in making proper diagnosis and referring patients to appropriate cancer centre. Only few patients received rituximab. Major factor behind the underutilization of rituximab was high cost of the drug. Response rate was high with more than half patients achieving CR. Interestingly; more CRs were seen in Binet stage C. Although, there was no significant difference in the response rate with FC or FCR regimen but PFS was better with the later. PFS with both regimens including FC and FCR were comparable with published data. Patients treated with chlorambucil or CVP were not included in survival analysis due to small number of patients in this group. Due to the same reason, FC and FCR were not compared for survival out comes.

## CONCLUSIONS

Based on our results, onset of CLL is in a relatively younger age group. Majority of the patients present with advanced stage disease and require treatment at the time of diagnosis. Fludarabine based regimens are affective. Addition of rituximab should be considered where available as it will lead to improved outcomes in CLL.

## References

[1] Gribben JG (2010). How I treat CLL up front. Blood.

[2] Binet JL, Auquier A, Dighiero G, Chastang C, Piguet H, Goasguen J (1981). A new prognostic classification of chronic lymphocytic leukemia derived from a multivariate survival analysis. Cancer.

[3] Rai KR, Sawitsky A, Cronkite EP, Chanana AD, Levy RN, Pasternack BS (1975). Clinical staging of chronic lymphocytic leukemia. Blood.

[4] Abrisqueta P, Pereira A, Rozman C, Aymerich M, Giné E, Moreno C (2009). Improving survival in patients with chronic lymphocytic leukemia (1980-2008):the Hospital Clinic of Barcelona experience. Blood.

[5] Dighiero G, Maloum K, Desablens B, Cazin B, Navarro M, Leblay R (1998). Chlorambucil in indolent chronic lymphocytic leukemia. French cooperative group on chronic lymphocytic leukemia. N Engl J Med.

[6] Eichhorst B, Hallek M, Dreyling M (2009). On behalf of the ESMO Guidelines Working Group. Chronic lymphocytic leukemia:ESMO Minimum Clinical Recommendations for diagnosis, treatment and follow-up. Ann Oncol.

[7] Hallek M, Fischer K, Fingerle-Rowson G, Fink AM, Busch R, Mayer J (2010). Addition of rituximab to fludarabine and cyclophosphamide in patients with chronic lymphocytic leukaemia:a randomised, open-label, phase 3 trial. Lancet.

[8] Robak T, Jamroziak K, Gora-Tybor J, Stella-Holowiecka B, Konopka L, Ceglarek B (2010). Comparison of cladribine plus cyclophosphamide with fludarabine plus cyclophosphamide as first-line therapy for chronic lymphocytic leukemia:a phase III randomized study by the Polish Adult Leukemia Group (PALG-CLL3 Study). J Clin Oncol.

[9] Kay NE, Geyer SM, Call TG, Shanafelt TD, Zent CS, Jelinek DF (2007). Combination chemoimmunotherapy with pentostatin, cyclophosphamide, and rituximab shows significant clinical activity with low accompanying toxicity in previously untreated B chronic lymphocytic leukemia. Blood.

[10] Eichhorst B, Fink AM, Busch R, Kovacs G, Maurer C, Lange E (2014). Frontline chemoimmunotherapy with fludarabine (F), cyclophosphamide (C), and rituximab (R) (FCR) shows superior efficacy in comparison to bendamustine (B) and rituximab (BR) in previously untreated and physically fit patients (pts) with advanced chronic lymphocytic leukemia (CLL):final analysis of an international, randomized study of the German CLL Study Group (GCLLSG) (CLL10 Study). Blood.

[11] Goede V, Fischer K, Busch R, Engelke A, Eichhorst B, Wendtner CM (2014). Obinutuzumab plus chlorambucil in patients with CLL and coexisting conditions. N Engl J Med.

[12] Hillmen P, Robak T, Janssens A, Govindbabu K, Grosicki S, Mayer J (2013). Ofatumumab +chlorambucil versus chlorambucil alone in patients with untreated chronic lymphocytic leukemia (CLL):results of the phase III study complement 1 (OMB110911). Blood.

[13] Hallek M, Stahel RA, Greil R (2005). ESMO Minimum Clinical Recommendations for diagnosis, treatment and follow-up of chronic lymphocytic leukemia. Ann Oncol.

[14] Hallek M, Cheson BD, Catovsky D, Caligaris-Cappio F, Dighiero G, Döhner H (2008). Guidelines for the diagnosis and treatment of chronic lymphocytic leukemia:a report from the International Workshop on Chronic Lymphocytic Leukemia updating the National Cancer Institute–Working Group 1996 guidelines. Blood.

[15] Shanafelt TD, Geyer SM, Kay NE (2004). Prognosis at diagnosis:integrating molecular biologic insights into clinical practice for patients with CLL. Blood.

[16] Chemotherapeutic options in chronic lymphocytic leukemia:a meta-analysis of the randomized trials (1999). CLL Trialists’ Collaborative Group. J Natl Cancer Inst.

[17] Dotan E, Aggarwal C, Smith MR (2010). Impact of Rituximab (Rituxan) on the Treatment of B-Cell Non-Hodgkin’s Lymphoma. Pharmacy and Therapeutics.

[18] Woyach JA, Ruppert AS, Heerema NA (2011). Chemoimmunotherapy With Fludarabine and Rituximab Produces Extended Overall Survival and Progression-Free Survival in Chronic Lymphocytic Leukemia:Long-Term Follow-Up of CALGB Study 9712. J Clin Oncol.

[19] Nunes AA, da Silva AS, Souza KM, Koury Cde N, de Mello LM (2015). Rituximab, fludarabine, and cyclophosphamide versus fludarabine and cyclophosphamide for treatment of chronic lymphocytic leukemia:A systematic review with meta-analysis. Crit Rev Oncol Hematol.

[20] Parikh SA, Rabe KG, Kay NE, Call TG, Ding W, Schwager SM (2014). Chronic lymphocytic leukemia in young (≤55 years) patients:a comprehensive analysis of prognostic factors and outcomes. Haematologica.

